# Influence of different arm movement strategies on dynamic balance performance and joint kinematics in healthy young adults

**DOI:** 10.3389/fspor.2025.1688698

**Published:** 2025-12-15

**Authors:** Johanna Lambrich, Katharina Borgmann, Mathew W. Hill, Thomas Muehlbauer

**Affiliations:** 1 Division of Movement and Training Sciences/Biomechanics of Sport, University of Duisburg-Essen, Essen, Germany; 2 Institute of Biomechanics and Orthopaedics, German Sport University Cologne, Cologne, Germany; 3 School of Psychology and Vision Sciences, University of Leicester, Leicester, United Kingdom

**Keywords:** postural control, reaching, walking, inertial-sensor-system, upper body strategy, task difficulty

## Abstract

**Background:**

Behavioural data indicate the existence of a complementary “upper body strategy” for postural control, especially during challenging dynamic balance tasks; however, information about joint kinematics associated with this strategy is limited, leaving a gap in our understanding of the underlying movement patterns.

**Objective:**

The objective was to investigate the influence of free vs. restricted arm movement strategies on balance outcomes and joint kinematics during challenging dynamic balance tasks.

**Methods:**

Dynamic balance performance was assessed in 25 healthy young adults (13 females, mean age: 24.5 ± 3.1 years) using the Y Balance Test–Lower Quarter (YBT–LQ) and the 3-m Beam Walking Backward task with two difficulty levels (beam width: 4.5 cm *vs.* 3.0 cm). Participants performed the balance assessments under two different arm positions: free and restricted arm movements. Reach distance, step number, and joint range of motion (ROM) were compared between test conditions.

**Results:**

Free compared to restricted arm movements led to significantly greater joint ROM, particularly in upper body segments such as the shoulder (*p* ≤ .001 to.002). Increased task difficulty also resulted in higher ROM across conditions (e.g., shoulder joint: *p* = .040 to.043), especially during free arm movements.

**Conclusions:**

The increased joint ROM with free arm movements, particularly under increased task difficulty indicates an active engagement of the upper body segments during dynamic postural control. This highlights the importance of free arm movements to enhance balance control through greater joint mobility.

## 1 Introduction

Postural control refers to the ability to attain, maintain or regain a balanced state in any posture or performing any activity (1)*.* A “lower body strategy” consisting of the ankle and hip strategy is postulated for the control of static balance tasks such as quiet standing (2, 3). Specifically, the *ankle strategy* minimises body sway by moving the entire body as a single-segment inverted pendulum with counteractive torques at the ankle joints (4, 5). In contrast, the *hip strategy* moves the body as a double-segmented inverted pendulum with counterphase motion at the ankle and hip joints (2, 6). In addition, recent studies (7, 8) suggest the existence of an “upper body strategy”, which occurs in particular during dynamic balance tasks such as walking. Empirical support for this strategy is drawn from studies showing that walking performance improves when arm movements were allowed compared to when they were restricted (9–12). For example, da Silva Costa et al. (12) showed that walking distance and step speed increased when balance beam walking (length: 4 m) was performed with free compared to restricted arm movements, indicating improved gait behaviour in this specific task. In addition, the performance enhancements during walking with free arm movements were still observed when the level of task difficulty was increased (i.e., reduction of beam width).

Despite these findings, the majority of the available studies (9–12) only used behavioural data such as the number of steps and walking distance/speed, from which only indirect conclusions can be drawn about the existence of an “upper body strategy”. However, additional kinematic analyses of angular parameters (e.g., angle position, angle velocity) make it possible to directly visualise the use of an “upper body strategy”. For instance, Wdowski et al. (13) reported increased joint range of motion (ROM) and joint movement variability in the pelvis when children performed a lateral jump-landing task with free vs. restricted arm movements. Furthermore, Rosenblum et al. (8) demonstrated that restricting arm movement while walking changes upper body kinematics and decreases dynamic stability. This emphasises the importance of free arm movement for stability in adults. These findings emphasise the importance of free arm movement for stability and highlight the relevance of kinematic analyses to capture upper-body contributions during dynamic balance tasks such as walking, offering deeper insights beyond behavioural outcomes alone.

Against the background outlined above, behavioural data in addition to kinematic data were recorded in the present study using an inertial-sensor-system during the execution of dynamic balance tasks (with diverging levels of task difficulty) under two different arm positions (free *vs*. restricted arm movements). While previous studies have examined the effects of different arm movement strategies on balance performance or kinematic outcomes in isolated tasks or specific populations (8, 13), the present study extends this work by combining multiple dynamic tasks of varying difficulty level with segmental kinematic analysis in healthy young adults. This provides a more comprehensive understanding of upper body contributions to human postural control. The objective of this study was to investigate how arm movements influence balance performance and joint kinematics during challenging dynamic tasks in young healthy adults. We hypothesised that free compared to restricted arm movements would improve balance performance (e.g., increased step number) and would elicit kinematic changes that primarily affect the upper body segments (i.e., significantly larger shoulder and elbow joint ROM) and with less pronounced effects on the lower body. Further, it was assumed that the increase in task difficulty would lead to a deterioration in dynamic balance performance (less during free arm movements) and result in an increase in joint ROM (more during free arm movements).

## 2 Material and methods

### 2.1 Participants and estimation of sample size

Estimation of sample size was conducted from previous studies (7, 13) that have investigated changes in balance performance and joint kinematics and when arm movements were free or restricted. G*Power software version 3.1.9.7 (14) was used and revealed that a minimum of 24 participants would be required to identify statistically significant differences in balance and kinematic outcomes between different arm movement strategies (input parameters: effect size [*f*] = .25, significance level [*α*] = .05, power [1-*β*] = .80, correlation among repeated measures = .50). Consequently, 25 healthy young adults (13 females; age: 24.6 ± 3.1 years; body mass: 73.7 ± 13.5 kg; body height: 178.1 ± 8.7 cm; body mass index: 23.1 ± 2.9 kg/m^2^) were recruited from the host institution's student population. All participants were free of musculoskeletal dysfunction, neurological impairment, or orthopaedic disorder, and reported normal or corrected-to-normal vision. Prior to the start of the study, all participants gave their written informed consent. The study was conducted in accordance with the Declaration of Helsinki and the human ethics committee at the University of Duisburg-Essen, Faculty of Educational Sciences, which approved the study protocol (approval number: EA-PSY10/17/07102017).

### 2.2 Experimental procedure

**Figure 1:**
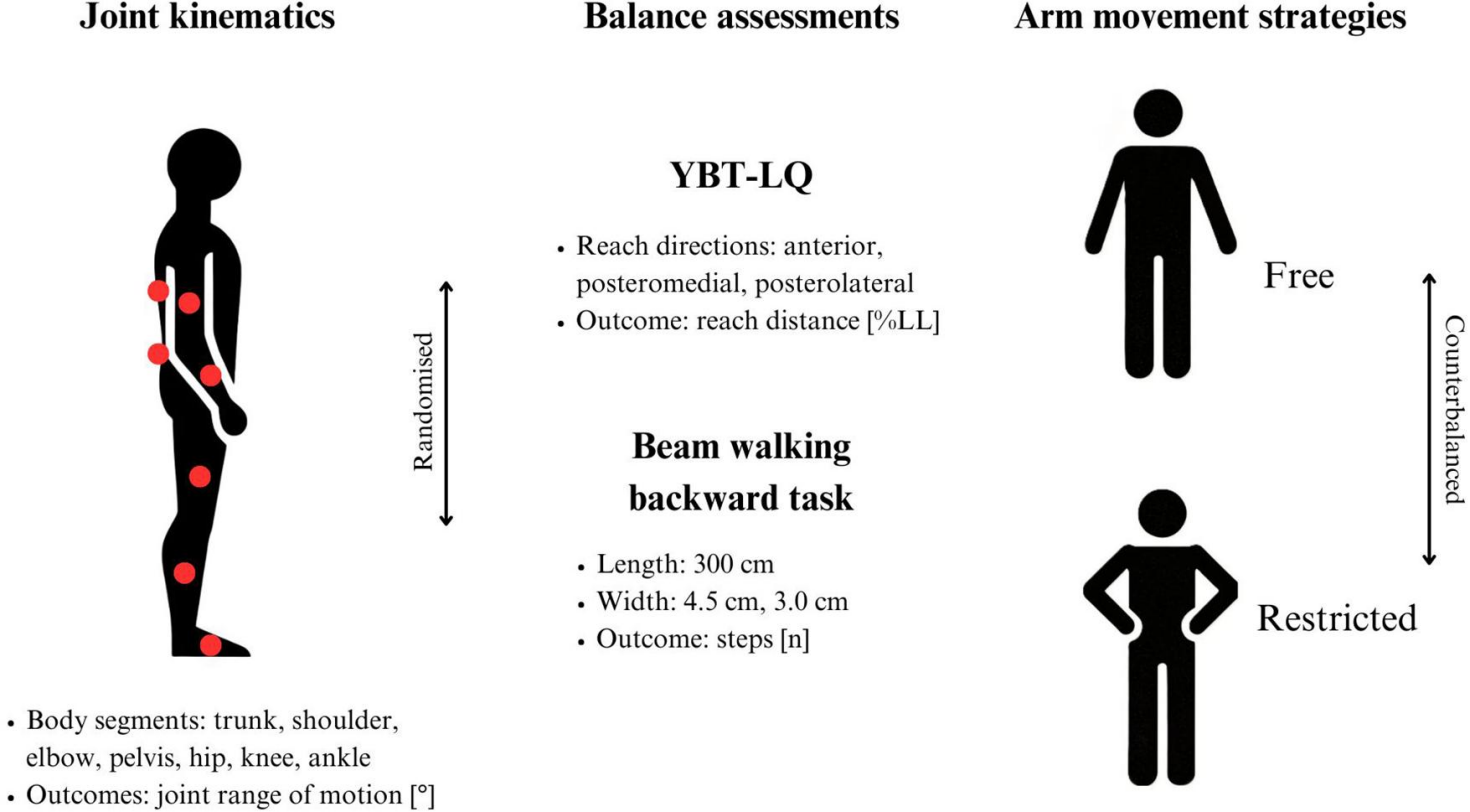
Schematic diagram of the experimental procedure showing the assessments (i.e., joint kinematics and balance performance) and test conditions (e.g., free vs. restricted arm movements). YBT–LQ, Y Balance Test–Lower Quarter.

A single-group repeated-measures design was used to assess differences in balance and kinematic outcomes using two arm positions (Figure 1). At the beginning, participants received verbal instructions about the experimental procedure. Thereafter, a standardised warm-up protocol was conducted that consisted of three minutes of rope skipping and two minutes of stretching. Subsequently, the participants randomly performed the Y Balance Test–Lower Quarter (YBT–LQ) and the 3-m Beam Walking Backward task (beam width: 4.5 cm *vs.* 3.0 cm) in two counterbalanced blocks, i.e., (1) free arm movements (i.e., arms free to move) and (2) restricted arm movements (i.e., hands placed on the hips). Participants completed the three reach directions of the YBT–LQ in a fixed order (i.e., starting with the anterior [AT] direction, followed by the posteromedial [PM] and posterolateral [PL] directions). The order of the two balance tasks (YBT–LQ and beam walking) and the two beam widths (4.5 cm vs. 3.0 cm) was randomized using computer-generated sequences. The order of arm movement conditions (free *vs.* restricted) was counterbalanced across participants using a list generated by an independent researcher.

### 2.3 Assessment of kinematic outcomes

A camera-free, portable three-dimensional inertial measurement unit (MyoMotion inertial-sensor-system, Noraxon, Scottsdale, AZ, USA) was used to collect kinematic data. Noraxon MyoResearch software was applied for data acquisition (sampling frequency: 100 Hz). Sensor placement followed the recommendations stated in the MyoMotion user manual (15) and is shown in Figure 1. A total of seven sensors were placed on each participant on the following body segments: upper thorax, pelvis, upper arm, forearm, thigh, tibia, and foot. From this, angles for the trunk, shoulder, elbow, pelvis, hip, knee, and ankle were determined for different movement directions. In terms of the trunk angle, the inertial sensors were placed on the upper thorax (below C7 and aligned with the spinal column) and on the pelvis (in the sacral region). The trunk angle was defined as the angle formed by the line connecting these two sensors and the horizontal plane. Validity of the MyoMotion inertial-sensor-system for movement assessment has been shown in a previous study (16). The kinematic analysis was limited to the dominant body side (determined via self-report) because it is biomechanically more active and more representative of typical movement patterns (17). Prior to each measurement session, a static calibration was performed according to the MyoMotion user manual. Participants stood in an upright neutral posture (feet shoulder-width apart, arms resting alongside the body, gaze directed forward) for 5 s to define zero positions for all joints.

### 2.4 Assessment of dynamic balance outcomes

Dynamic balance was assessed using the YBT–LQ. The Y-Balance test kit (Functional Movement Systems®, Chatham, USA) was used for this purpose. The test kit consists of a centralised platform with three attached pipes representing the AT, PM, and PL reach directions. Each pipe is marked in 1.0-cm increments to measure reach distances and is equipped with a moveable reach indicator. Participants were instructed to move the reach indicator as far as possible in the AT direction with their non-dominant leg while standing on the centralised platform with their dominant leg. The protocol was repeated for the PM and PL directions. Each participant completed three practice trials, followed by three data-collection trials for each leg and reach direction. A trial was considered invalid if any of the following occurred: (1) loss of balance, such as the non-dominant leg touched the ground, (2) lifting the standing leg off the platform, (3) stepping onto the sliding pipe with the non-dominant leg, (4) sliding the reach indicator improperly, or (5) removing the hands from the hips in the restricted arm movement condition. The YBT–LQ has demonstrated “excellent” reliability (18). The maximal reach distance (cm) per reach direction was normalised to the participants' leg length (LL) to account for individual differences in limb proportions and used for further analyses.

Dynamic balance was additionally measured using a walking backward task consisting of a 300 cm long wooden beam with two different beam widths: 4.5 cm (represents a low difficulty level) and 3.0 cm (represents a high difficulty level). Participants wore their own sport shoes and were instructed to walk with as much control as possible backward without stepping off the beam at a self-selected speed from the beginning to the end of the balance beam, whereby a maximum of eight steps was used for further analyses. Each participant completed one practice trial, followed by two data-collection trials. A trial was considered invalid and was repeated if any of the following occurred: (1) loss of balance (e.g., stepping off the beam) or (2) removing the hands from the hips in the restricted arm movement condition. The number of steps per trial was registered and the mean of the two data-collection trials was used for further analyses.

### 2.5 Data analysis

Kinematic data were processed using MATLAB software version R2022b (The MathWorks Inc., Natick, MA, USA). The joint angle data were low-pass filtered using a 4th-order zero-lag Butterworth filter at 10 Hz and time-normalised to 5,000 data points, enabling consistent comparisons between trials of different durations. The ROM was then calculated for each trial as the difference between the maximum and minimum joint angles. Subsequently, the ROM was computed for 18 joint movements. Positive angles indicated that the trunk was extended and bent to the right, the shoulder was moved forwards (anteversion), abducted and externally rotated, the elbow was flexed, the pelvis was externally rotated and tilted anterior and upwards, the hip was flexed, abducted, and externally flexed, the knee was flexed, bent inwards (valgus), and externally rotated, and the ankle was dorsiflexed, abducted, and the inside lifted (inversion). Contrary, negative angles indicated that the trunk was flexed and bent to the left, the shoulder was moved backwards (retroversion), adducted and internally rotated, the elbow was extended, the pelvis was internally rotated and tilted posterior and downwards, the hip was extended, adducted, and internally flexed, the knee was extended, bent outwards (varus), and internally rotated, and the ankle was plantarflexed, adducted, and the outside lifted (eversion).

### 2.6 Statistical analysis

Statistical analysis was conducted using Jeffreys's Amazing Statistics Program (JASP) version 0.17.3 (Amsterdam, The Netherlands) and RStudio version 2024.12.0 (R Foundation for Statistical Computing, Vienna, Austria). Descriptive data are reported as group mean values ± standard deviations (*SD*). The assumptions of normality (assessed with the Shapiro–Wilk Test) and homogeneity of variance/sphericity (assessed with Mauchly's Test) were confirmed prior to performing parametric analysis. For the YBT–LQ, univariate analysis of variance (ANOVA) was conducted to determine differences in balance and kinematic outcomes between free arm and restricted arm movement strategies. For the Beam Walking Backward task, a series of two-way ANOVAs were performed to investigate the within-subject effects of arm movement condition (free *vs.* restricted) and task difficulty (low *vs.* high). In case of significant main effects or arm movement × task difficulty interactions, *post-hoc* analyses using Bonferroni-adjusted *α* levels were applied to determine the location of any difference. For the ANOVAs, we calculated the effect size using partial eta-squared (*η*_p_^2^), which was categorised as small (.02 ≤ *η*_p_^2^ ≤ .12), medium (.13 ≤ *η*_p_^2^ ≤ .25), or large (*η*_p_^2^ ≥ .26). For the *post-hoc* analyses, Cohen's *d* was computed and classified as trivial (0 ≤ *d* ≤ .19), small (.20 ≤ *d* ≤ .49), moderate (.50 ≤ *d* ≤ .79), or large (*d* ≥ .80). The significance level was *a priori* set at *p* < .05.

## 3 Results

### 3.1 Balance outcomes

Table 1 shows the results for all balance outcomes for the free and restricted arm movement conditions. For the YBT–LQ, reach distances in the PM (*p* = .003, *η*_p_^2^ = .17) and PL (*p* = .004, *η*_p_^2^ = .16) directions were significantly larger with free compared to restricted arm movements, whereas no significant difference was observed for the AT reach direction (*p* = .101, *η*_p_^2^ = .06). Further, participants performed significantly more steps in the Beam Walking Backward task with free compared to restricted arm movements, irrespective of beam width (4.5 cm: *p* = .005, *η*_p_^2^ = .16; 3.0 cm: *p* < .001, *η*_p_^2^ = .25). Further analyses revealed that increased task difficulty was accompanied with a significant deterioration in balance performance that was large sized during walking with free (*t* = 4.779, *p* < .001, *d* = .96) and restricted (*t* = 4.692, *p* < .001, *d* = .94) arm movements.

**Table 1:** Descriptive statistics (mean ± SD) for all balance outcomes with free compared to restricted arm movements.

Test/outcome	Free	Restricted	*p* (*η*_p_^2^)
YBT–LQ			
YBT–LQ: AT reach (%LL)	71.7 ± 7.2	68.5 ± 6.2	.101 (.06)
YBT–LQ: PM reach (%LL)	120.1 ± 9.6	112.8 ± 6.5	**.003** (**.17)**
YBT–LQ: PL reach (%LL)	116.1 ± 7.4	110.0 ± 6.6	**.004** (**.16)**
Beam walking backward task			
4.5 cm beam walk [steps]	7.7 ± 1.1	6.2 ± 2.2	**.005** (**.16)**
3.0 cm beam walk [steps]	6.3 ± 1.9	4.2 ± 1.7	**<.001 (.25)**

Bold values indicate statistically significant differences. Threshold values for the *η*_p_^2^ were.02 ≤ *η*_p_^2^ ≤ .12 (small),.13 ≤ *η*_p_^2^ ≤ .25 (medium), *η*_p_^2^ ≥ .26 (large). AT, anterior; LL, leg length; PL, posterolateral; PM, posteromedial; YBT–LQ, Y Balance Test–Lower Quarter.

### 3.2 Kinematic outcomes

#### 3.2.1 Y balance test–lower quarter

Table 2 illustrates the results for the joint ROM for the free and restricted arm movement conditions while performing the YBT–LQ. For the AT reach direction, the analysis revealed significantly greater joint ROM for the shoulder (anteversion/retroversion: *p* < .001, *η*_p_^2^ = .39; abduction/adduction: *p* < .001, *η*_p_^2^ = .23; external/internal rotation: *p* < .001, *η*_p_^2^ = .24) and elbow (flexion/extension: *p* = .015, *η*_p_^2^ = .12) during the free compared to the restricted arm movement condition. Concerning the PM reach direction, we observed significantly larger joint ROM for the shoulder (anteversion/retroversion: *p* < .001, *η*_p_^2^ = .68; abduction/adduction: *p* < .001, *η*_p_^2^ = .40) during the free vs. restricted arm movement condition. With regard to the PL reach direction, the analysis yielded significantly greater joint ROM for the trunk (flexion/extension: *p* < .001, *η*_p_^2^ = .26), shoulder (anteversion/retroversion: *p* < .001, *η*_p_^2^ = .74; abduction/adduction: *p* < .001, *η*_p_^2^ = .50; external/internal rotation: *p* = .002, *η*_p_^2^ = .19), elbow (flexion/extension: *p* = .016, *η*_p_^2^ = .11), and knee (flexion/extension: *p* = .023, *η*_p_^2^ = .10) during the free compared to the restricted arm movement condition.

**Table 2:** Descriptive (mean ± SD) and inference statistics for the joint range of motion while performing the Y balance test–lower quarter with free and restricted arm movements.

Joint	Movement	Anterior reach			Posteromedial reach			Posterolateral reach		
		Free	Restricted	*p* (*η*_p_^2^)	Free	Restricted	*p* (*η*_p_^2^)	Free	Restricted	*p* (*η*_p_^2^)
Trunk	Extension (+)/flexion (-)	41.3 ± 18.0	36.4 ± 18.7	.355 (.02)	69.4 ± 12.5	63.4 ± 11.6	.082 (.06)	74.6 ± 8.4	64.9 ± 8.5	**<.001 (.26)**
	Right (+)/left (-) bending	39.7 ± 58.9	24.2 ± 24.5	.230 (.03)	114.0 ± 107.4	96.4 ± 82.2	.519 (.01)	143.0 ± 80.9	116.6 ± 91.6	.286 (.02)
Shoulder	Anteversion (+)/retroversion (-)	71.7 ± 37.5	22.8 ± 23.4	**<.001 (.39)**	118.9 ± 41.7	24.3 ± 21.2	**<.001 (.68)**	111.6 ± 36.2	20.7 ± 14.6	**<.001 (.74)**
	Abduction (+)/adduction (-)	75.4 ± 44.8	21.7 ± 55.4	**<.001 (.23)**	118.2 ± 67.9	22.7 ± 50.0	**<.001 (.40)**	139.6 ± 88.6	16.2 ± 11.4	**<.001 (.50)**
	External (+)/internal (-) rotation	45.4 ± 23.1	19.7 ± 23.4	**<.001 (.24)**	54.7 ± 54.2	29.8 ± 59.3	.128 (.05)	74.1 ± 74.7	23.5 ± 17.1	**.002** (**.19)**
Elbow	Flexion (+)/extension (-)	38.2 ± 23.3	20.8 ± 25.3	**.015** (**.12)**	43.0 ± 34.9	31.9 ± 79.2	.526 (.01)	51.6 ± 64.0	19.1 ± 12.7	**.016** (**.11)**
Pelvis	Anterior (+)/posterior (-) flexion	13.6 ± 6.2	11.8 ± 4.5	.261 (.03)	23.9 ± 16.2	20.7 ± 8.0	.385 (.02)	49.3 ± 16.8	45.4 ± 15.0	.384 (.02)
	Upward (+)/downward (-) tilt	25.1 ± 13.2	21.3 ± 11.3	.286 (.02)	60.4 ± 40.9	51.6 ± 11.7	.303 (.02)	66.6 ± 42.7	53.3 ± 10.7	.136 (.05)
	External (+)/internal (-) rotation	10.8 ± 3.5	8.9 ± 3.3	.053 (.08)	17.1 ± 37.2	9.4 ± 3.2	.308 (.02)	23.6 ± 39.5	14.6 ± 4.7	.265 (.03)
Hip	Flexion (+)/extension (-)	62.4 ± 33.9	56.8 ± 29.8	.540 (.01)	107.1 ± 26.0	99.3 ± 17.5	.214 (.03)	112.5 ± 20.5	100.9 ± 21.5	.056 (.07)
	Abduction (+)/adduction (-)	23.8 ± 8.1	22.9 ± 11.1	.765 (.00)	26.9 ± 8.4	27.3 ± 10.3	.863 (.00)	22.6 ± 7.8	20.4 ± 6.8	.291 (.02)
	External (+)/internal (-) rotation	16.2 ± 5.9	17.1 ± 10.3	.684 (.00)	35.3 ± 54.6	22.4 ± 7.9	.246 (.03)	22.5 ± 8.9	21.0 ± 6.5	.526 (.01)
Knee	Flexion (+)/extension (-)	94.1 ± 30.6	85.7 ± 24.9	.296 (.02)	86.3 ± 14.3	78.7 ± 13.4	.060 (.07)	73.7 ± 12.8	65.5 ± 11.9	**.023** (**.10)**
	Valgus (+)/varus (-)	13.0 ± 6.6	12.4 ± 7.8	.782 (.00)	14.9 ± 6.8	14.8 ± 8.9	.975 (.00)	31.5 ± 8.9	30.1 ± 11.6	.628 (.00)
	External (+)/internal (-) rotation	17.6 ± 8.2	15.3 ± 6.6	.285 (.02)	16.8 ± 4.5	15.6 ± 3.8	.321 (.02)	23.0 ± 4.5	22.2 ± 6.0	.593 (.01)
Ankle	Dorsiflexion (+)/plantarflexion (-)	35.6 ± 7.4	34.4 ± 7.0	.546 (.01)	27.6 ± 7.5	27.6 ± 6.4	.961 (.00)	28.6 ± 6.7	28.0 ± 6.1	.748 (.00)
	Abduction (+)/ adduction (-)	16.5 ± 4.5	16.4 ± 6.0	.948 (.00)	18.9 ± 6.4	17.7 ± 4.3	.476 (.01)	18.9 ± 6.0	18.7 ± 4.9	.898 (.00)
	Inversion (+)/eversion (-)	15.1 ± 4.2	13.4 ± 4.8	.199 (.03)	14.6 ± 3.8	13.8 ± 4.1	.467 (.01)	16.9 ± 4.4	15.8 ± 4.5	.394 (.02)

Bold values indicate statistically significant differences. Threshold values for the *η*_p_^2^ were.02 ≤ *η*_p_^2^ ≤ .12 (small),.13 ≤ *η*_p_^2^ ≤ .25 (medium), *η*_p_^2^ ≥ .26 (large).

#### 3.2.2 Beam walking backward task

**Figure 2:**
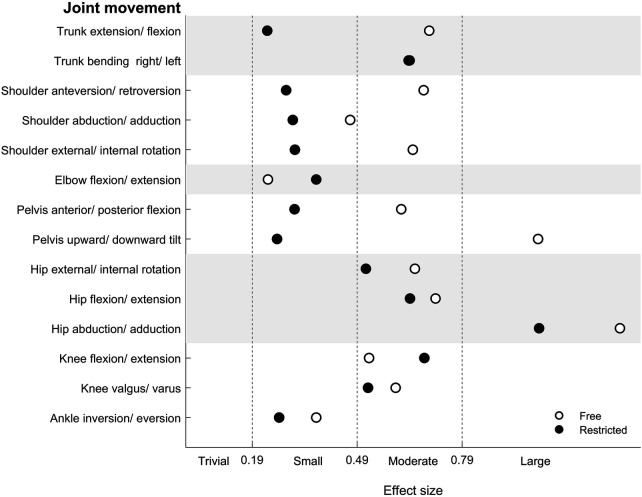
Effect sizes for the joint range of motion differences resulting from the statistical comparison of both beam widths (i.e., 3.0 cm vs. 4.5 cm) representing different levels of task difficulty by arm movement strategy (i.e., free vs. restricted).

Table 3 displays the results for the joint ROM for the free and restricted arm movement conditions while performing the Beam Walking Backward task. There was a significant main effect of arm condition for the shoulder (anteversion/retroversion: *p* < .001, *η*_p_^2^=.51; abduction/adduction: *p* < .001, *η*_p_^2^ = .59; external/internal rotation: *p* < .001, *η*_p_^2^ = .62) and elbow (flexion/extension: *p* < .001, *η*_p_^2^ = .43), indicating an increase in joint ROM from walking with restricted compared to free arm movements. Further, we observed a significant main effect of task difficulty for the trunk (extension/flexion: *p* < .001, *η*_p_^2^ = .12; right/left bending: *p* = .003, *η*_p_^2^ = .09), shoulder (anteversion/retroversion: *p* = .041, *η*_p_^2^ = .04; abduction/adduction: *p* = .040, *η*_p_^2^ = .04; external/internal rotation: *p* = .043, *η*_p_^2^ = .04), pelvis (anterior/posterior flexion: *p* = .002, *η*_p_^2^ = .10; upward/downward tilt: *p* = .010, *η*_p_^2^=.07), hip (flexion/extension: *p* < .001, *η*_p_^2^ = .13; abduction/adduction: *p* < .001, *η*_p_^2^ = .23; external/internal rotation: *p* = .003, *η*_p_^2^ = .09), knee (flexion/extension: *p* = .006, *η*_p_^2^=.08; valgus/varus: *p* = .029, *η*_p_^2^=.05), and ankle (inversion/eversion: *p* = .037, *η*_p_^2^ = .04), that is indicative of an increase in joint ROM when walking under more (beam width: 3.0 cm) compared to less (beam width: 4.5 cm) challenging conditions. Further analyses yielded that an increase in task difficulty was associated with a significant increase in joint ROM, particularly during free vs. restricted arm movements (i.e., in 11 out of 14 joint movements) (Figure 2). The arm movement × task difficulty interactions did not reach statistical significance.

**Table 3:** Descriptive (mean ± SD) and inference statistics for the joint range of motion while performing the beam walking backward task with free and restricted arm movements.

Joint	Movement	Beam width: 4.5 cm		Beam width: 3.0 cm		Arm (free *vs.* restricted)	Difficulty (low *vs.* high)	Arm × difficulty interaction
		Free	Restricted	Free	Restricted	*p* (*η*_p_^2^)	*p* (*η*_p_^2^)	*p* (*η*_p_^2^)
Trunk	Extension (+)/flexion (-)	37.3 ± 15.7	34.9 ± 13.7	48.0 ± 15.0	45.5 ± 15.1	.404 (.01)	**<.001 (.12)**	.982 (.00)
	Right (+)/left (-) bending	60.8 ± 31.6	65.9 ± 47.4	100.3 ± 51.9	81.3 ± 44.9	.437 (.01)	**.003** (**.09)**	.180 (.02)
Shoulder	Anteversion (+)/retroversion (-)	96.0 ± 50.6	24.0 ± 13.2	124.6 ± 62.6	30.1 ± 18.7	**<.001 (.51)**	**.041** (**.04)**	.181 (.02)
	Abduction (+)/adduction (-)	91.9 ± 37.4	16.2 ± 9.7	117.8 ± 61.3	21.2 ± 15.1	**<.001 (.59)**	**.040** (**.04)**	.159 (.02)
	External (+)/internal (-) rotation	68.6 ± 24.7	24.7 ± 10.1	80.3 ± 24.2	28.7 ± 12.4	**<.001 (.62)**	**.043** (**.04)**	.309 (.01)
Elbow	Flexion (+)/extension (-)	59.2 ± 26.9	22.9 ± 13.7	63.7 ± 21.9	29.3 ± 18.4	**<.001 (.43)**	.193 (.02)	.813 (.00)
Pelvis	Anterior (+)/posterior (-) flexion	23.5 ± 8.8	31.2 ± 18.5	35.8 ± 12.9	36.7 ± 12.7	.118 (.03)	**.002** (**.10)**	.218 (.02)
	Upward (+)/downward (-) tilt	28.7 ± 15.1	30.1 ± 22.2	38.7 ± 13.6	38.3 ± 17.5	.879 (.00)	**.010** (**.07)**	.790 (.00)
	External (+)/internal (-) rotation	17.8 ± 12.1	23.6 ± 39.8	24.8 ± 11.0	25.1 ± 9.3	.495 (.00)	.340 (.01)	.537 (.00)
Hip	Flexion (+)/extension (-)	50.2 ± 15.6	53.1 ± 17.4	62.3 ± 16.0	66.2 ± 17.6	.315 (.01)	**<.001 (.13)**	.881 (.00)
	Abduction (+)/adduction (-)	26.0 ± 7.3	29.4 ± 8.1	35.5 ± 8.2	37.3 ± 8.8	.116 (.03)	**<.001 (.23)**	.653 (.00)
	External (+)/internal (-) rotation	26.1 ± 9.9	26.5 ± 7.4	31.6 ± 6.1	32.1 ± 12.0	.800 (.00)	**.003** (**.09)**	.965 (.00)
Knee	Flexion (+)/extension (-)	53.4 ± 14.6	56.2 ± 12.0	61.5 ± 17.0	64.6 ± 14.2	.319 (.01)	**.006** (**.08)**	.954 (.00)
	Valgus (+)/varus (-)	16.9 ± 10.7	15.6 ± 5.2	20.5 ± 9.4	19.7 ± 8.6	.539 (.00)	**.029** (**.05)**	.903 (.00)
	External (+)/internal (-) rotation	27.2 ± 6.2	27.5 ± 5.3	30.2 ± 6.2	29.1 ± 7.9	.760 (.00)	.081 (.03)	.587 (.00)
Ankle	Dorsiflexion (+)/plantarflexion (-)	46.7 ± 8.2	44.4 ± 8.1	55.5 ± 30.0	51.2 ± 32.7	.474 (.01)	.090 (.03)	.831 (.00)
	Abduction (+)/ adduction (-)	29.7 ± 6.7	31.3 ± 6.0	32.3 ± 9.6	30.9 ± 8.9	.939 (.00)	.465 (.01)	.345 (.01)
	Inversion (+)/eversion (-)	39.8 ± 12.0	38.0 ± 7.3	51.3 ± 30.1	46.7 ± 34.1	.502 (.00)	**.037** (**.04)**	.761 (.00)

Bold values indicate statistically significant differences. Threshold values for the *η*_p_^2^ were.02 ≤ *η*_p_^2^ ≤ .12 (small),.13 ≤ *η*_p_^2^ ≤ .25 (medium), *η*_p_^2^ ≥ .26 (large).

## 4 Discussion

Overall, the results of the present study show that dynamic balance performance was consistently better under free compared to restricted arm movement conditions, across all test items. Furthermore, higher task difficulty resulted in poorer performance, as indicated by a smaller number of successfully completed steps during the beam walking backward task. Additionally, the shoulder and elbow joint ROM was greater during free vs. restricted arm movements, further emphasising the role of the upper body in balance control. We investigated the influence of different arm movement strategies on balance outcomes and joint kinematics during dynamic balance tasks in healthy young adults. The assumption that free compared to restricted arm movements would lead to better balance performances and larger joint ROM (particularly in the upper body segments) was confirmed and is consistent with previous studies (7, 10, 13, 19, 20). In terms of balance performance, Sogut et al. (20) studied young adults (mean age: 22.7 ± 1.9 years) and reported significantly greater YBT–LQ reach distances for the PM and PL reach direction when the arms moved freely compared to restricted arms movements. Moreover, Muehlbauer and colleagues (10) examined young adults (mean age: 24.7 ± 3.0 years) and stated a significantly greater number of steps during beam walking backwards for the arms free compared to the arms restricted test condition. With regard to joint kinematics, Wdowski et al. (13) investigated children (mean age: 10.1 ± 1.6 years) and stated increased joint ROM in the pelvis when performing a lateral jump-landing task with free compared to restricted arm movements. Further, Boström and colleagues (7) asked young adults (mean age: 24.27 ± 3.01 years) to walk forward on a balance beam and reported that torque amplitude of the upper body joints were significantly greater when the arms moved freely compared to hands placed on the thighs. The improved dynamic balance performances and the increased joint ROM with free vs. restricted arm movements can be explained by several mechanical factors. Specifically, extending the arms shifts body mass further away from the axis of rotation, which increases the moment of inertia and slows down angular velocity (9). Furthermore, free arm movements can be used to generate restoring torques which reduces the body's angular momentum (21). In addition, arm movements can act as counterweight to move the centre of mass away from the direction of instability (22). In light of the aforementioned findings and our present results, it is plausible to argue that free arm movements represent an “upper body strategy” that allows people to actively use their upper body to counteract postural instability. This strategy may act in addition to lower body mechanisms and thereby contribute to maintain postural stability (23). From a practical perspective, these findings can be used for people involved in injury- or fall-preventive programs to perform balance exercises first with free arm movements. Especially when introducing new exercises, allowing free arm movements may help individuals to successfully accomplish the task and provide greater safety as well. For example, a recently published study (24) showed that the time in balance was longer when acquiring a stabilometer task with free than restricted arms. In the later course of balance exercise, restriction of arm movements can be used, resulting in a decrease in the degree of freedom and evoking adaptations in the postural control system leading to optimised dynamic balance performance.

The hypothesis that the increase in task difficulty would (i) lead to a deterioration in dynamic balance performance, that would be less pronounced during free compared to restricted arm movements and (ii) result in an increase in joint ROM, particularly during free arm movements was confirmed and is in line with previous work (7, 11, 12, 19). For example, Muehlbauer et al. (10) tested young adults (mean age: 24.7 ± 3.0 years) using the Beam Walking Backward task. They found greater performance decrements when walking across the narrow vs. wide beam, in particular when arm movements were restricted. In addition, da Silva and colleagues (12) studied older adults (age range: 65–76 years) that walked forward on 10.0-, 8.0-, and 6.0-cm wide beams. Distance walked and step speed was reduced but trunk acceleration was increased with decreasing beam width (i.e., increased task difficulty). Furthermore, Boström et al. (7) asked young adults (mean age: 24.3 ± 3.0 years) to walk over 6.0-, 4.5- and 3.0-wide beams. The authors reported that the contribution of upper body movements to dynamic balance performance (i.e., torque amplitude/variation) significantly increased when the task difficulty increased (i.e., beam width decreased). These studies and the present work support the notion that the supportive influence of free arm movements on postural control appears to be stronger with increasing task difficulty (19, 25). Thus, an increase in task difficulty at later stages of balance training, in addition to the restriction of the arm movements, can be easily introduced as a task constraint by practitioners to progressively induce further adaptation processes in the postural control system.

The present study has several limitations that should be considered when interpreting the results. First, healthy young adults were investigated, which limits the generalisability of our results to younger, older or health-impaired populations. Second, the kinematic analysis focused exclusively on the dominant side of the body, which does not consider potential bilateral differences or compensatory strategies of the non-dominant side. Third, kinematic data was collected using inertial sensors on seven body segments, which limits the level of detail and analysis of fine motor movements. Our analysis focused exclusively on joint kinematics derived from IMU measurements. While this approach allows detailed characterization of movement patterns across joints, it does not capture underlying kinetic contributions, such as joint torques, that may influence center-of-mass control. Consequently, only indirect indications of possible compensation strategies can be inferred.

## 5 Conclusions

We examined the effect of different arm movement strategies on dynamic balance performance and joint kinematics in healthy young adults. This study provides a detailed kinematic description of joint movements in the upper and lower body during dynamic balance tasks, during free and restricted arm movement. This offers complementary insights to previous behavioral research. Precisely, free compared to restricted arm movements led to improved balance performance and increased joint ROM, confirming that humans actively use their upper body to counteract postural instability and indicating the existence of a complementary “upper body strategy” for dynamic balance tasks. The stabilising effects of free arm movements were greater for challenging task conditions, which implies that their contributing role increases with increasing task difficulty.

## Data Availability

The raw data supporting the conclusions of this article will be made available by the authors, without undue reservation.
